# From design to clinic: Engineered peptide nanomaterials for cancer immunotherapy

**DOI:** 10.3389/fchem.2022.1107600

**Published:** 2023-01-17

**Authors:** Jing-Jing Du, Zhenhong Su, Haoyi Yu, Sanhai Qin, Dongyuan Wang

**Affiliations:** ^1^ Hubei Key Laboratory of Kidney Disease Pathogenesis and Intervention, College of Medicine, Hubei Polytechnic University, Huangshi, China; ^2^ Department of Pharmacy, Union Hospital, Tongji Medical College, Huazhong University of Science and Technology, Wuhan, China; ^3^ Hubei Province Clinical Research Center for Precision Medicine for Critical Illness, Wuhan, China

**Keywords:** peptide, vaccines, nanomaterials, cancer immunotherapy, clinic

## Abstract

Immunotherapy has revolutionized the field of cancer therapy. Nanomaterials can further improve the efficacy and safety of immunotherapy because of their tunability and multifunctionality. Owing to their natural biocompatibility, diverse designs, and dynamic self-assembly, peptide-based nanomaterials hold great potential as immunotherapeutic agents for many malignant cancers, with good immune response and safety. Over the past several decades, peptides have been developed as tumor antigens, effective antigen delivery carriers, and self-assembling adjuvants for cancer immunotherapy. In this review, we give a brief introduction to the use of peptide-based nanomaterials for cancer immunotherapy as antigens, carriers, and adjuvants, and to their current clinical applications. Overall, this review can facilitate further understanding of peptide-based nanomaterials for cancer immunotherapy and may pave the way for designing safe and efficient methods for future vaccines or immunotherapies.

## Introduction

### Peptide ligands as versatile cancer antigens to activate immune systems

Cancer immunotherapy (CIT) is a validated and critically important approach for treating patients with cancer. It utilizes the body’s immune system to target and eliminate cancer cells with enduring anti-tumor responses to prevent cancer metastasis and recurrence (Rosenberg et al., 2004; Pardoll, 2012). In human bodies, two major immune systems—the innate and adaptive immune systems—successfully recognize and eliminate hidden cancer niches (Zhang et al., 2019). Thus, cancer vaccines must be potent enough to be recognized and then activate the related immune response. Peptide-based vaccines have attracted much attention and interest in cancer immunotherapy, owing to their high affinity, easy preparation, and safety, and they have been proven to have significant efficacy in inhibiting tumor growth and preventing tumor relapse and metastasis (Zhang et al., 2019; Cai et al., 2020; Malonis et al., 2020). After immunization, antigen-presenting cells (APCs) usually capture and internalize the peptide-based cancer vaccines. Then, they present antigenic fragments on the surface MHC class I/MHC class II (MHC I/MHC II) alleles of APCs, thereby stimulating the activation of CD4 T cells or cytotoxic T lymphocytes. Activated T cells release cytokines and induce cytotoxic T lymphocyte (CTL) responses, and CD4 T cells promote B-cell proliferation and IgG production (Feng et al., 2016). Although peptide vaccines have showed promising anti-cancer effects *in vivo*, their low stability and penetration properties have hindered their further clinical application (Kuai et al., 2017).

Advanced biomaterials and drug delivery systems have become useful tools for improving CIT potency while reducing toxic side effects in a safer, more controlled manner, and for adjusting the tumor immune environment to increase the response to immunotherapies (Duan et al., 2019; Aikins et al., 2020; Gong et al., 2021). Peptide-based nanomaterials are promising agents in cancer therapy, serving as target ligands, carriers, antigens, or adjuvants in these nanomedicines with good efficiency and safety (Jiang et al., 2019; Cai et al., 2020; Dersh et al., 2021; O'Neill et al., 2021). Peptides are designed as versatile cancer vaccines due to their multifunctional properties. For instance, peptides can modify the pharmacokinetics and biodistribution of nanomedicines for immunotherapy and can efficiently deliver immunotherapeutic agents to tumor tissues (Zhang et al., 2010; Zhao et al., 2013). Meanwhile, peptides themselves participate in activating the tumor immune response. In this mini-review, we briefly summarize recent progress in peptide antigen design, nanomaterials for peptide antigen delivery, peptide-based nanomaterials as carriers or adjuvants to enhance the effect of CITs, and the clinical applications of these peptide-based nanomaterials in cancer therapy. Finally, we discuss the challenges that peptide-based vaccines meet in further clinical cancer immunotherapy.

### Self-assembled peptide-based materials used as cancer antigens

The epitope for developing peptide-based therapeutic cancer vaccines is a short amino sequence derived from tumor antigens with immunogenicity and HLA allele compatibility (Shemesh et al., 2021). Peptide-targeting antigens can be classified into two categories: tumor-associated antigens (TAAs) overexpressed in tumor tissues and tumor-specific antigens (TSAs) specifically expressed in tumor tissues but not in normal tissues and which include the mutated protein, cancer testis antigens, and virus-related antigens (Garcia-Soto et al., 2017; Vitiello and Zanetti, 2017; Peng et al., 2019; Liu et al., 2021). TAA-targeting peptide vaccines have been widely developed from various cancer-related proteins such as VEGFR (Okuyama et al., 2013), HER2/neu (Knutson et al., 2001), CEA (Antonilli et al., 2016), MUC1 (Antonilli et al., 2016), survivin (Ciesielski et al., 2010), EGFR (Chen et al., 2018), and FR (folate receptor), and they have been applied in various cancers such as lung cancer (Mami-Chouaib et al., 2002), breast cancer (Peres Lde et al., 2015), liver cancer (Shen et al., 2017), melanoma (Ruiter et al., 1991), leukemia (Sugiyama, 2002), and ovarian cancer (Antonilli et al., 2016). For instance, the peptide vaccine (LEEKKGNYVVTDHC) derived from epidermal growth factor receptor variant III (EGFRvIII) has showed stronger immune responses and longer median progression-free survival in patients with newly diagnosed EGFRvIII-expressing glioblastoma in a phase 2 trial (Sampson et al., 2010; Chen et al., 2018). The FR-derived peptide vaccines E39 (FR-α 191−199) and E41 (FR-α 245−253) are efficiently presented to CD8^+^ T-cells and have showed potent anticancer effects in ovarian-cancer animals and good safety but weak immune response in ovarian cancer patients in a phase II clinical trial (Chianese-Bullock et al., 2008). TSA-based vaccines derived from mutation-derived epitopes can be presented as foreign antigens on the surfaces of tumor cells and APCs, which could increase the frequency of recognition by cytotoxic T lymphocyte (CTL) precursors and induce strong and fast CTL activation (Herlyn and Birebent, 1999; Gubin et al., 2015). These peptide vaccines usually use multiple cancer peptides with an appropriate, individualized selection to complement pre-existing host immunity, which induces stronger and more rapid antitumor immunity in comparison with inoculation of conventional peptide vaccines (Shemesh et al., 2021). Thus, TSA-derived peptide vaccines have great potential for personalized cancer therapy.

**FIGURE 1:**
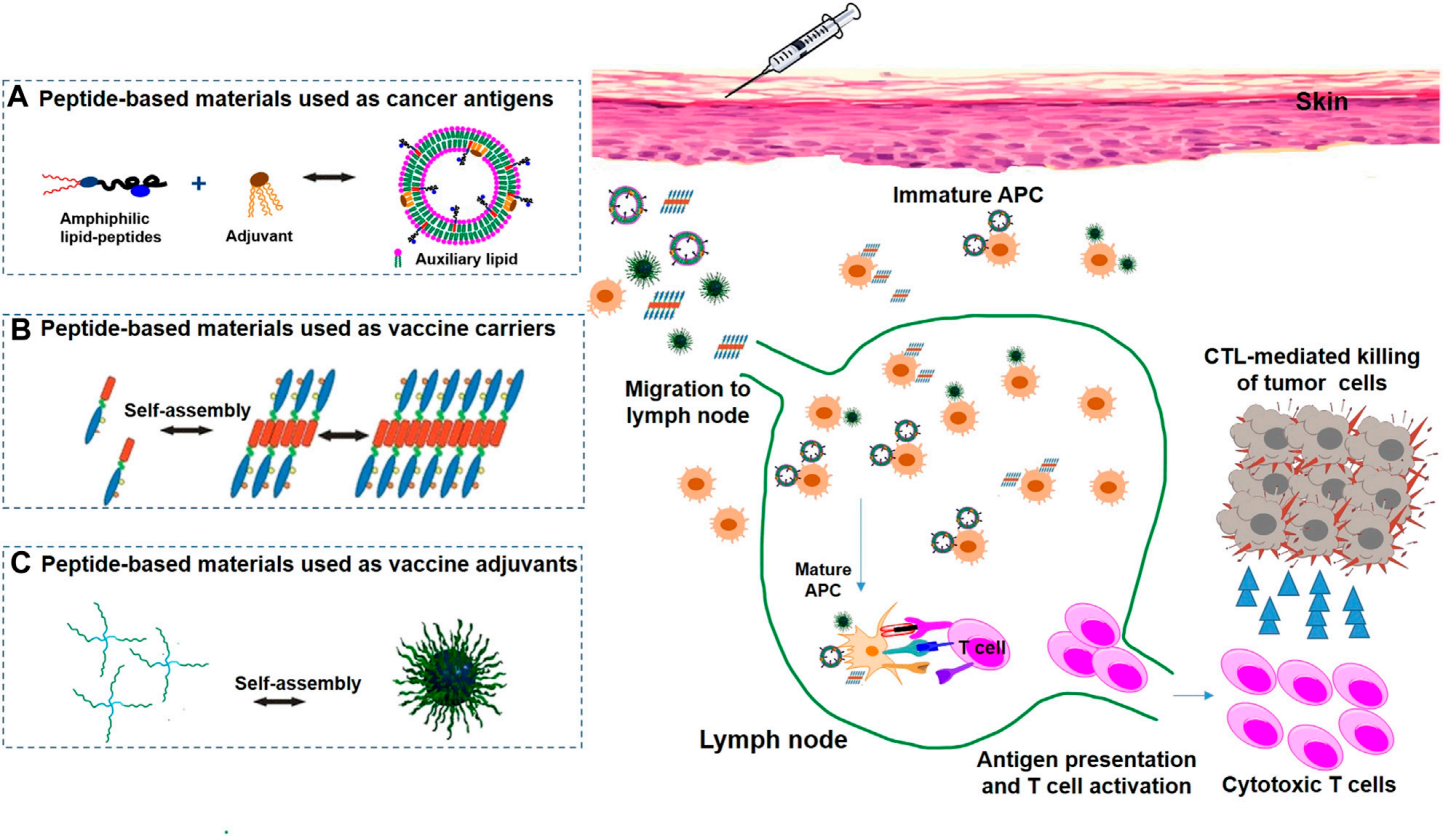
Peptide-based nanomaterials used in cancer immunotherapy via various delivery systems, including peptide-based nanomaterials as antigens **(A)**, carriers **(B)** and adjuvants **(C)**.

Peptide vaccines can be classified into two groups according to their activation functions: one group can activate the innate immune system by interacting with tumor-associated macrophages (TAMs), dendritic cells (DCs), neutrophils, and natural killer (NK) cells, and the other group can activate the adaptive immune system by interacting with T cells and B cells (Figure 1A) (Rosenberg et al., 1998; Zhang et al., 2019). Peptide vaccines targeting TAMs are aimed at blocking protumoral M2-TAMs activities impeding the recruitment of macrophages to tumors and switching M2-TAMs into protumoral M1-TAMs (Tang et al., 2013; David, 2017). To prolong the presentation of an MHC class I-restricted self-peptide on DCs, the peptide vaccines were loaded in DCs to activate T cells for over 24 h, protecting immunized mice from tumor progression and suppressing lung metastases (Wang and Wang, 2002). HLA-A24 peptide- (CEA652-) loaded DC vaccines could prevent further tumor growth and decrease the levels of carcinoembryonic antigen (CEA) in serum (Ueda et al., 2004). NK cells are cytotoxic lymphocytes able to recognize stressed cells and rapidly respond to tumor formation without the aid of antibodies and MHC (Caligiuri, 2008). Chapel et al. 2017 reported that an HLA-C*06:02-presented peptide could bind to the NK-activating receptor KIR2DS1, which was sufficient for the activation of primary KIR2DS1 (+) NK cells (cytotoxic CD8^+^ T cells are responsible for killing cancer cells, a process that can be blocked by inhibitory receptor ligands like PD-L1 or PD-L2 expressed on cancer cells) (Thomas and Massague, 2005). Li et al. 2018 identified a PD-L1-targeted peptide (SGQYASYHCWCWRDPGRSGGSK) with high affinity, which was able to retard tumor growth in mice to a larger degree than a PD-L1 antibody (56% vs. 71%, respectively), demonstrating its high therapeutic efficiency. B cell targeting peptide vaccines are derived from immunogenic proteins containing B cell epitopes that can induce B cells to create antibodies (Yuen et al., 2016). Another method is the use of B cell peptide mimics that can directly bind to tumor-specific cellular receptors to block downstream signals and induce cancer death (Kaumaya, 2015). Recently, B cell-based peptide cancer vaccines have been developed targeting HER2/neu receptors (Wiedermann et al., 2010), EGFR (Zhu et al., 2013), and others, which can produce specific IgG antibodies and have demonstrated strong antitumor activity in mice. Based on these successes, the HER-2 or EGFR targeting peptide vaccines have been tested in cancer patients in phase I or II clinical trials (Riemer et al., 2005; Wiedermann et al., 2010).

Many clinical trials have shown that peptide-based vaccines are safe and effective in various cancer types. However, further studies are needed to identify and evaluate the peptide vaccines in different cancer patients, who will derive the greatest benefit from this approach, and to optimize the therapeutic protocols.

### Peptide-based materials serve as potent vaccine carriers

Peptide nanomaterials are considered to be promising carrier structures and have been proved to be capable of delivering peptide antigens and immunoadjuvants with good stability and high cargo loading capacity (Koutsopoulos et al., 2009; Lyu and Azevedo, 2021). Many one-dimensional filaments or nanofibers based on self-assembly of peptides and peptide derivatives could be intertwined to form hydrogels or nanoparticles, facilitating the controlled delivery of peptides and adjuvants (Lyu and Azevedo, 2021).

Hydrogel has been used as a good delivery system, which has great prospects in the combination of antigen delivery and therapeutics to induce potent CTLs (Figure 1B) (Binaymotlagh et al., 2022). Collier’s research group developed vaccines using a Q11 self-assembling domain (Ac-QQKFQFQFEQQ-Am) from chicken egg ovalbumin (OVA323-339), applied for the linkage of a MUC1-derived peptide (Rudra et al., 2010). Q11 is a self-assembly peptide that can aggregate into nanofibers in salt-containing aqueous environments (Rudra et al., 2012). These vaccines induced robust antibodies, and their antibodies could effectively recognize breast cancer cells. As an improvement of the strategy, a fully synthetic self-adjuvanting vaccine with a self-assembling Q11 domain was designed to aggregate into fibrils and show multivalent B-cell epitopes under mild conditions, further enhancing the immunogenicity of the TAAs (Huang et al., 2012). Recently, novel self-assembling peptide epitopes (SAPEs) were successfully applied to vaccine delivery systems and were generated using a peptide (Ac-AAVVLLLW-COOH) or a thermosensitive polymer poly (N-isopropylacrylamide (pNIPAm)) attached covalently to different peptide antigens. Compared to sham-treated mice, tumor-bearing mice inoculated with SAPEs could inhibit tumor growth and prolong survival time (Rad-Malekshahi et al., 2017).

The self-assembly of antigenic peptides into hydrogels is a feasible strategy to augment anti-tumor immunity. For instance, Leach and others reported the use of the hydrogel of the self-assembling multidomain peptide (MDP) (K_2_(SL)_6_K_2_) for encapsulation of stimulator of interferon genes-(STING-) agonist cyclic dinucleotides (CDNs; STINGel) (Leach et al., 2018). STINGel is a peptide hydrogel that prolongs overall survival in a challenging murine model of head and neck cancer. Recently, Leach et al. loaded a drug-mimicking peptide hydrogel (L-NIL-MDP) with a STING agonist (CDN), resulting in 4- and 20-fold slower drug release than with commercially available hydrogels (Leach et al., 2021). Consequently, L-NIL-MDP+CDN is an effective, bioactive carrier material for cancer immunotherapies and successfully increased the survival of tumor-burdened mice. Li and coworkers took a further step by developing a tumor cell-derived cancer vaccine (PVAX) for postsurgical immunotherapy (Wang et al., 2018). PVAX was prepared by encapsulating BRD4 inhibitor (JQ-1) and indocyanine green (ICG) co-loaded tumor cells through a hydrogel matrix, which could simultaneously elicit antitumor immunity and block the PD-L1/PD-1 checkpoint to prevent tumor recurrence and metastasis. In order to expand the application of hydrogels, (Xiao et al. 2022) proposed a ternary hydrogel composed of polyvinyl alcohol (PVA), polyethylenimine (PEI), and magnesium ions, which can upregulate PD-L1 expression and promote the polarization of M1-like macrophages, thus achieving excellent immunomodulation function. Additionally, (Wang et al. 2022) developed a hydrogel (CM@Gel) to encapsulate MnO_2_ nanosheets and the vascular-disrupting CA4P, which not only enhanced immunotherapy but also avoided the possibility of vascular disruption during systemic administration.

Virus-like particles (VLPs) are constructed by self-assembly of viral envelope proteins and can be used as potent delivery systems for cancer vaccines (Cai et al., 2020) (Wang et al., 2021). For example, (Storni et al. 2004) demonstrated that VLP vaccination was capable of inducing high levels of peptide-specific cytotoxic T cell responses and eradicating established solid fibrosarcoma tumors. CpGs and p33 peptide were packaged together into VLPs, enhancing their stability and improving their immunogenicity. Additionally, carcinoembryonic antigen (CEA) has been chosen as a promising target structure in the construction of colorectal cancer vaccines. One study indicated that an oral PV-CEA pseudovirus vaccine induced high CEA-specific CTL responses and efficiently inhibited tumor growth (Huang et al., 2005). Interestingly, Jemon’s research group designed a rabbit hemorrhagic disease virus-based VLP (RHDV-VLP) targeting HPV16-positive tumors. The RHDV-VLP vaccine was comprised of the Th epitope PADRE on the internal surface of the VLP and an MHC I-restricted peptide from the HPV16 E648-57 on the external surface. E6-RHDV-VLP-PADRE not only improved the overall efficacy of the cancer treatment but also prolonged the survival rate of mice with HPV-based tumors (Jemon et al., 2013).

### Peptide-based materials used as cancer vaccine adjuvants

Molecular adjuvants are highly relevant to the development of cancer immunotherapy and are widely applied in vaccination to activate immune pathways, thereby enhancing antitumor immune responses (Coffman et al., 2010). Many adjuvants are essential for cancer immunotherapy, such as aluminum salts (Awate et al., 2013), oil-in-water emulsions (Guy, 2007), low-toxicity QS-21 (Li and Li, 2020), toll-like receptor (TLR) agonist (Zhou et al., 2018), NKT-cell agonist (Li et al., 2022), stimulator of interferon gene (STING) (Van Herck et al., 2021), and other biological materials. However, the participation of these molecular adjuvants makes understanding the mechanisms and securing regulatory approval of cancer vaccines challenging. Thus, the research and development of approaches for self-adjuvanting or adjuvant-free delivery systems has become a hot field in the treatment of cancers (Jiang et al., 2017).

Due to the advantages of self-assembled materials, such as low immunogenicity, compatibility, versatility, and multivalence, they have been explored as adjuvants of vaccines and have made much exciting progress (Koirala et al., 2022). The most successful self-assembly adjuvant is the bacterial lipopeptide Pam_3_Cys-Ser-(Lys)_4_ (Pam_3_CSK_4_) (Figure 1C) (Basto and Leitão, 2014). Boons and coworkers developed several antitumor vaccines with Pam_3_CSK_4_ as a built-in adjuvant, which elicited stronger CTL responses (Ingale et al., 2007). In addition, Payne’s group showed that a multicomponent vaccine incorporating Pam_3_CSK_4_ as an adjuvant and a T-helper epitope of the tetanus toxoid protein covalently linked to a MUC1 glycopeptide was significantly more immunogenic and elicited remarkable levels of IgG antibodies (Wilkinson et al., 2011). Meanwhile, they further developed a novel conjugate vaccine with macrophage-activating lipopeptide (MALP2) as an immunoadjuvant, which could provoke robust humoral immune responses, since MALP2 is a potent activator of TLR2/6 in immune cells (McDonald et al., 2014).

More interestingly, fibroblast-stimulating lipopeptide 1 (FSL-1, Pam_2_CGDPKHPKSF) has also been used as an immunoadjuvant to prime TLR2/6 and TLR2/1 signaling pathways (Zhou et al., 2018). To improve the immunogenicity of TAAs, Zhao et al. reported that a novel type of MUC1-FSL-1 conjugate as a self-adjuvanting (glyco)lipopeptide cancer vaccine could induce T-cell mediated responses capable of killing tumor cells (Liu et al., 2016). GFFY-based adjuvants as another series of self-assembly adjuvants were successfully developed for the construction of vaccines. For example, Yang’s research group reported that an antigen-mixing supramolecular hydrogel of a D-tetra-peptide (G^D^F^D^F^D^Y) and a naphthylacetic acid-modified derivate (Nap-G^D^F^D^F^D^Y) were capable of serving as attracting adjuvants to prime both humoral and cellular immune responses (Luo et al., 2017; Yang et al., 2018). Additionally, Yang et al. proposed a new strategy, using the low-toxicity cholesterol-modified antimicrobial peptide (AMP) DP7 (DP7-C), which has dual functions as a carrier and an immune adjuvant, which effectively improves the efficiency of antigen presentation and enhances the efficacy of individualized cancer immunotherapy (Zhang et al., 2020). Considering these advantages, these self-assembly adjuvants are potential strategies for cancer immunotherapy.

### Clinical applications of peptide-based nanoparticles for cancer immunotherapy

Currently, numerous peptide-based therapeutic cancer vaccines have been tested in cancer patients and have achieved significant clinical benefits. The clinical peptide vaccines were mainly from tumor-associated antigens (TAAs) and tumor-specific antigens (TSA) and targeted malignant cancers including melanoma (Kawakami et al., 1994), lung cancer (Mami-Chouaib et al., 2002), breast cancer (Wiedermann et al., 2010), and leukemia (Schwartzentruber et al., 2011) in phase I, II, and III clinical trials. The targeted peptide vaccines can induce the immune response and are well tolerated in cancer patients in phase I clinical trials. However, in phase II and III clinical trials, these peptide vaccines had limited therapeutic effects (Malonis et al., 2020). For example, the HER2-derived peptide vaccine NeuVax can stimulate specific CD8^+^ CTLs that recognize and destroy HER2-expressing cancer cells (Brossart et al., 1998). In a phase III clinical trial, NeuVax did not impact breast cancer recurrence as compared to a placebo (Mittendorf et al., 2019). Clinical treatment with NeuVax combined with trastuzumab is ongoing for HER2-positive breast cancer patients (NCT02297898 and NCT01570038), which may pave the way for the clinical application of peptide vaccines. Single-peptide vaccines may have limited immune response; thus, multiple peptide vaccines in combination are being investigated in ongoing clinical trials. The phase I clinical trial using five-FR-α peptide (FR30, FR56, FR76, FR113, and FR238) admixed with GM-CSF, called TIPV200, was tested in ovarian and breast cancer patients (Kalli et al., 2018). TIPV200 was well tolerated, and more than 90% of the patients slowly developed immunity within five months, which persisted for at least a year. Currently, phase II clinical trials of TPIV200, alone or combined with the cancer immunotherapy drug durvalumab, are ongoing in ovarian cancer patients (Kalli et al., 2018). TAA-derived peptide vaccines had limited immune response, especially for patients with low-expressing targets. TSA-derived peptide vaccines can be regarded as personalized peptide vaccines (PPVs), which can cause an increased frequency of recognition by CTL precursors, thus inducing strong and fast CTL activation (Shemesh et al., 2021). Thus, TSAs are attractive for personalized cancer immunotherapy with good efficiency and tolerance (Kimura et al., 2017). The first randomized phase II trial of PPVs in prostate cancer was reported in 2010 and showed that PPV, plus a low dose of estramustine phosphate (EMP), led to longer median progression-free survival (PFS) and overall survival than did EMP alone (Noguchi et al., 2010). Another clinical trial also verified that PFS was significantly longer in the PPV-plus-dexamethasone group than in the dexamethasone in castration-resistant prostate cancer (CRPC) patients (Yoshimura et al., 2016). To minimize tumor immune escape caused by antigen loss, peptide-based vaccines in clinical trials often combine multiple targets with multiple epitopes. To further improve therapeutic efficacy, peptide-based vaccines are used in combination with other immunotherapies or chemotherapeutics in clinical trials, where they have demonstrated superior efficacy in cancer therapy (Kimura et al., 2017).

Besides exploring effective epitope peptides for activating CTLs, nanomaterials have been exploited to optimize the efficiency of the immune response of the epitope peptide to improve its clinical application (Kuai et al., 2017; Aikins et al., 2020). Liposomes were the first class of therapeutic nanoparticles to be approved for cancer treatment and still represent a large proportion of clinical-stage nanotherapeutics (Large et al., 2021). Liposome-based vaccines could co-deliver peptides and adjuvants to promote their delivery to lymphoid organs, an approach which shows great clinical potential for cancer immunotherapy (Miyabe et al., 2014). Other nanostructures based on amphiphilic dendrimers (Chahal et al., 2016) and cross-linked block copolymer micelles (Wang et al., 2020) are also acceptable strategies. Currently, only one peptide-based material (Pam_3_Cys) is being used as an adjuvant in the clinic for Lyme disease vaccines (Steere et al., 1998). Most peptide nanomaterial-based cancer vaccines are under preclinical study, and improvements in their stability and permeability may also facilitate the availability of peptide materials in clinical cancer immunotherapies. For example, liposomal vaccines whose lipid membranes have good fluidity and permeability (Senior and Gregoriadis, 1982) can allow maximum drug release. In addition, cholesterol and DSPC can enhance the structural stability of liposomes and enable maintenance of liposomal size within two months (Maritim et al., 2021).

## Discussion

Cancer immunotherapy is regarded as the most promising method of cancer therapy, as it pursues prolonged tumor regression by restoring and normalizing the immune surveillance of patients (Yang, 2015). However, the poor immunogenicity of cancer cells, low tumor infiltration of immune cells, and the presence of multiple immune checkpoints lead to low benefits in cancer immunotherapy (Topalian et al., 2012). Due to its tunability and multifunctionality, nanotechnology can achieve specific delivery of multiple cargos into tumors and immune cells to improve the efficacy and safety of drugs (Zhang et al., 2019; Gong et al., 2021). Peptide materials have received increasing interest, as they are easy to manufacture and have excellent safety profiles and relatively low costs, and they have showed great potential for clinical cancer therapy (Jiang et al., 2019). In this review, we summarize the design of peptide antigens, the use of peptide-based materials as carriers for specific delivery, the use of adjuvants to help stimulate the immune response, and current clinical studies of these peptide-based vaccines. Peptide antigens can significantly enhance systemic immune responses, generate effective immune cell responses in tumor tissue, and inhibit malignant tumor metastasis or recurrence. Additionally, these peptide vaccines are relatively safe and well tolerated in clinical trials. Peptide-based delivery systems can deliver therapeutic cargoes into specific cell lines with low toxicity and high therapeutic effect. Meanwhile, peptide-based adjuvants are effective enough to help stimulate the immune response. Despite the noteworthy advances in peptide-based vaccines for cancer therapy, peptide-based vaccines have unfortunately failed in clinical trials due to the lack of continuous and high immune response levels. As TAAs and MHC-I vary widely among patients and tumors, it is necessary to create personalized vaccines for individual patients. Peptide antigens should be widely screened to ensure that they are potent enough to activate sustained immune responses and inhibit cancer growth in patients; metabolomic, proteomic, and genomic screening of natural products can be used to identify bioactive peptides (Shemesh et al., 2021). Moreover, in order to be clinically translated, these peptide-based materials must be stable for large-scale production and storage (Zhang et al., 2019). The combination of peptide-based vaccines with other therapies is also a good direction for improving clinical outcomes for cancer patients, such as chemotherapy, radiotherapy (RT), biological agents, and immune checkpoint inhibitors (Liu et al., 2021). As the field of immuno-oncology improves, we sincerely expect the synergistic development of peptide materials with other immunotherapies to yield innovative strategies for tumor immunotherapy.
